# A Qualitative Exploration of Stroke Survivors' Experiences of Using a Stroke Helpline

**DOI:** 10.1111/hex.14141

**Published:** 2024-07-11

**Authors:** Muneeba T. Chaudhry, Alana B. McCambridge, Esminio I. I. Rivera, Scott William, Peter Stubbs, Arianne Verhagen, Caleb Ferguson

**Affiliations:** ^1^ Discipline of Physiotherapy, Graduate School of Health University of Technology Sydney Sydney New South Wales Australia; ^2^ Centre for Chronic & Complex Care Research, School of Nursing, Blacktown Hospital University of Wollongong & Western Sydney Local Health District Sydney New South Wales Australia

**Keywords:** helpline, self‐care, self‐management, stroke, telehealth, transitional care

## Abstract

**Background:**

StrokeLine is a stroke‐specific helpline used by stroke survivors and their families in Australia to access professional support. There has been little research exploring stroke survivors' experiences of using helplines and their perceived impact on their stroke recovery.

**Aim:**

The aim of this study is to explore the reasons prompting stroke survivors to call StrokeLine and their experiences and to describe the perceived impact of calling StrokeLine on their recovery.

**Methods:**

An exploratory descriptive qualitative study was undertaken using thematic analysis of data collected through semi‐structured interviews of stroke survivors between December 2020 and May 2022. Participants were recruited using purposive sampling. Interviews were conducted via audio‐recorded Zoom conference calling and transcribed verbatim for thematic analysis.

**Results:**

A total of eight callers (four men and women women) participated, with the time since stroke ranging from 3.5 months to 5 years. Four major themes were identified, including 17 sub‐themes. Key themes included (1) factors prompting use of StrokeLine; (2) experience of using StrokeLine; (3) perceived impact of using StrokeLine; and (4) conceptualising StrokeLine service provision.

**Conclusions:**

Participants perceived their experience of contacting StrokeLine as having a positive impact on their stroke recovery, leaving them feeling empowered and motivated to self‐manage their condition.

**Patient or Public Contribution:**

Stroke survivors with lived experience influenced the conceptualisation of this study through conversations with consumers and the Stroke Foundation. Eight stroke survivors were involved as participants in the research study.

## 1 Introduction

In Australia alone, the number of people living with the long‐term sequelae of stroke will increase to an estimated 700,000 people by 2032 [1]. The physical and psychosocial impacts of stroke are significant, with care needs evolving at every stage of recovery [2, 3]. As with most chronic conditions, facilitating self‐management in stroke is essential to empower survivorship and reduce the burden on health systems [4, 5]. Currently, there remains a low level of satisfaction with information provided after discharge from formal care in acute settings, particularly around long‐term management of disability and accessing follow‐up services [6, 7]. Stroke survivors who require support to navigate between transitions of care through the healthcare system report a feeling of abandonment [2, 3]. Telehealth‐based care may be helpful in providing effective self‐management support to stroke survivors across the continuum of stroke recovery [8, 9, 10].

Telephone‐based services, such as helplines, are able to provide access to timely support [11]. Specialised helplines led by qualified health professionals exist globally for chronic conditions such as cancer, mental health and heart disease, helping to facilitate self‐management of care [4]. An international study exploring the rationale, experience and impact of seeking care using telephone‐based support for cancer found that callers were able to better understand their situation, facilitating further engagement with other cancer services [12]. In addition, a Swedish rheumatology helpline was effective at enabling constructive dialogue and providing motivational support for callers who had problems obtaining answers from other care settings [13]. Ensuring that telehealth‐based care is relevant to the needs of the user is important to ensure that services remain sustainable in the future [14]. Globally, Stroke helplines exist in the United States of America and as part of the NHS in the United Kingdom. However, research into helplines for stroke survivors remains scarce.

In Australia, StrokeLine operates nationwide and is a free inbound phone‐delivered stroke support service provided by the Stroke Foundation, a not‐for‐profit organisation (and Australia's peak body and national voice of stroke), providing resources for those affected by stroke. The service operates from a single site (based in Melbourne, Australia) between routine business hours on weekdays and is staffed by qualified health professionals from a nursing or allied health background who can be contacted via phone, email or social media. StrokeLine staff offer advice and support to stroke survivors and their families, health professionals and the public. The role of telehealth‐based support services for stroke survivors is not well understood and the impact of telephone‐based stroke care needs further investigation. To date, research into stroke helplines is extremely limited, focusing only on user characteristics and a limited understanding of how they are used [15, 16]. It is important to understand service provision from the perspective of key users, particularly to ensure that health services remain relevant to the user's needs [17]. There is no research that has explored caller experiences of contacting a stroke helpline and little is known about how stroke survivors perceive their encounter with such a service.

The aims of this study were to (i) explore the reasons that prompt stroke survivors to use a stroke helpline and (ii) examine the perceived impact of the encounter on their stroke journey.

## 2 Methods

### 2.1 Design

This study followed a qualitative exploratory design and was guided by the consolidated criteria for reporting qualitative research (COREQ) [18]. Data were collected using semi‐structured interviews with stroke survivors between December 2020 and June 2022 to explore their experiences of using StrokeLine, a telehealth‐based support service. Informed consent from participants was obtained verbally using a scripted checklist at the beginning of each interview. Ethical approval for this study was granted by the Human Research Ethics Committee at the University of Technology Sydney (No.: ETH20‐5088).

### 2.2 Participants and Recruitment

#### 2.2.1 Inclusion Criteria

Participants must have had a stroke at any time, be 18 years or older, had contact with StrokeLine in the past 3 months, be able to communicate in English, have access to and be able to use conference calling and be able to provide verbal consent. Exclusion criteria were as follows: People who had contacted StrokeLine and were health professionals, members of the general public and those not affected by stroke including carers and family members.

Stroke survivors with aphasia were not explicitly excluded from this study, as suitability for inclusion would be determined on a case‐by‐case basis in conjunction with an expert speech pathologist familiar with qualitative interviewing strategies for people with aphasia.

The Stroke Foundation in Australia assisted with recruitment of participants for the study, through study advertisements included in monthly Stroke Foundation newsletters, on Stroke Foundation and authors' social media channels (e.g., Twitter and Facebook) and online forum posts associated with the Stroke Foundation's EnableMe (stroke survivor platform) service for stroke survivors and their carers. StrokeLine staff also sent prescripted emails directly to callers who were stroke survivors in two recruitment cycles, capturing only callers who had called StrokeLine in the preceding 3 months.

Potential participants contacted the researchers and an information sheet about the study was emailed to them before being screened over the phone for inclusion. If a participant was deemed eligible, an interview time with the researcher was scheduled within a week.

### 2.3 Procedures

Before each interview, the participant informed the researchers of their age, gender, location, time since stroke, if they lived alone and whether they identified as an Aboriginal or Torres Strait Islander. Due to the COVID‐19 pandemic and recruitment of participants across Australia, all interviews with participants were conducted online using audio‐recorded Zoom conference calling. Interviews were conducted by a qualified member of the research team lasted from 25 to 40 min. The interviewer (M.T.C.) was a female, accredited exercise physiologist (AEP) experienced in working clinically with stroke survivors during their recovery and rehabilitation. The interviewer was also supervised and mentored in the conduct of qualitative interviewing by a more experienced member of the research team (C.F.). A standardised interview guide was followed to ensure that key questions remained consistent across participants. Interviews were semi‐structured and directed by participant narratives around their experiences of using StrokeLine. The interview guide is supplied as supplementary materials.

Purposive sampling was used to ensure that characteristics of the participants included were representative of the diverse population spread in Australia and the trajectory of recovery in stroke. Time since stroke was categorised as either acute, subacute or chronic as defined by Bernhardt et al. [19]. Geographic location was categorised as either metro, rural or remote based on the Australian Statistical Geography Standard (ASGS)—Remoteness Area framework [20].

### 2.4 Data Analysis

Each interview was audio‐recorded and later transcribed verbatim for analysis by M.T.C. All identifying features of the data were removed before being shared amongst the research team. Written field notes were kept to aid with transparency of data collection. Interview transcripts were sent to participants for review to validate the data collected [21].

Data from transcribed interviews were analysed using a data‐driven inductive latent approach to thematic analysis. Transcripts were read multiple times to aid familiarisation and coded into categories using Excel [22]. Next, data were broadly coded by two independent assessors (M.T.C. and E.I.I.R.); themes were agreed by consensus. Themes were reviewed, defined and named [22]. For each theme, subthemes were also named and identified. The two assessors discussed the analysis to ensure that participant views were interpreted through multiple perspectives at each step [20]. Data were coded as soon as each interview was transcribed and checked. The criteria for trustworthiness in qualitative research first outlined by Guba and Lincoln (1989) were used to ensure the methodological rigour and integrity of the findings [23]. In particular, field notes and reflections during and after each interview were maintained to better establish an audit trail and add to the dependability of the findings [24].

## 3 Results

### 3.1 Study Population

**Figure 1:**
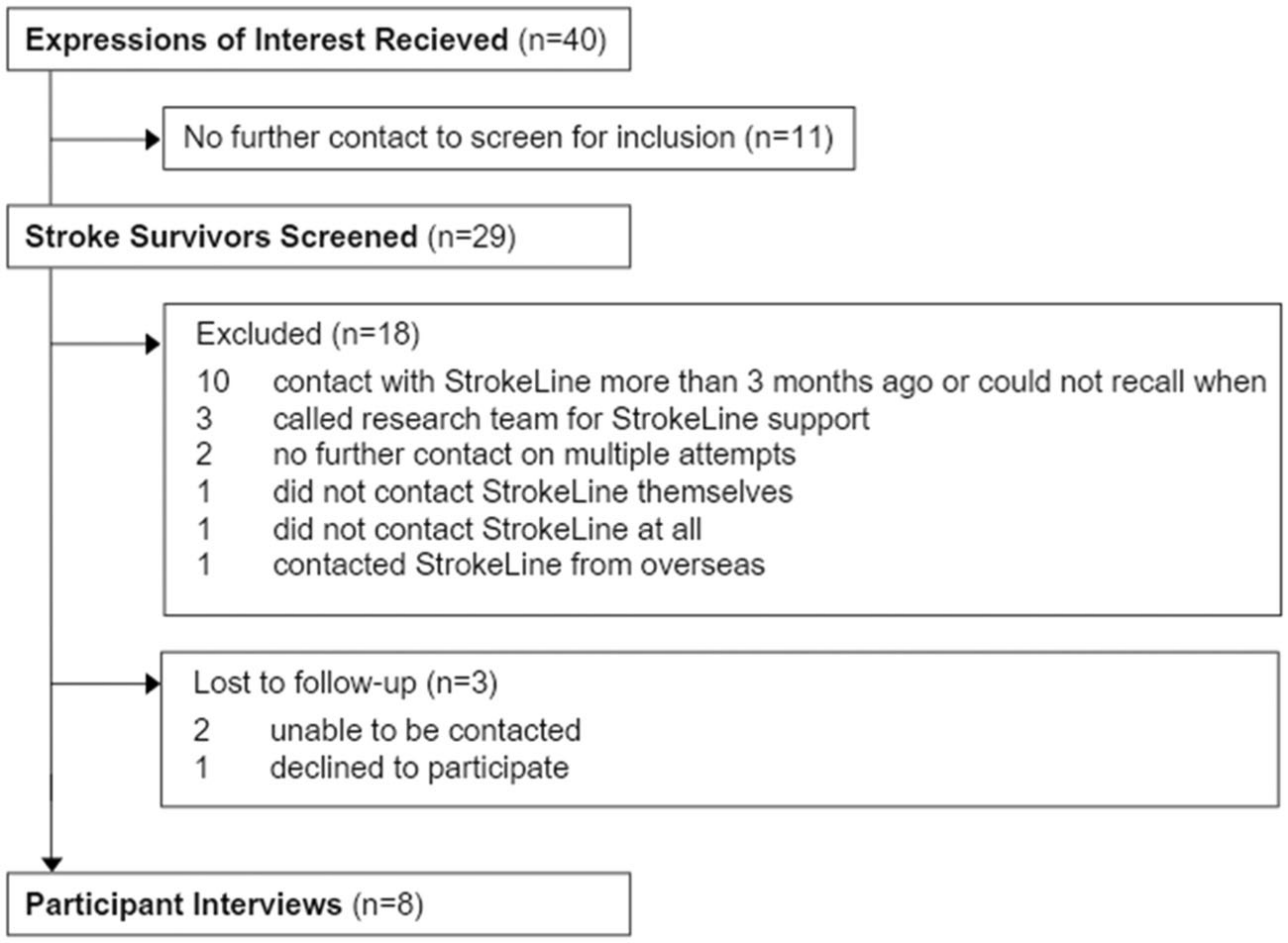
Participant flowchart.

Between December 2020 and June 2022, 40 stroke survivors expressed an interest in participating, with 29 screened for eligibility. Eight callers (four men and four women), ranging in ages from 28 to 82 years, were included. Figure 1 shows the flow of participants throughout the study. Participants' demographics and key characteristics are presented in Table 1.

**Table 1:** Characteristics of participants.

Participant	Age at the time of interview	Gender	Time since stroke	Stroke recovery stage^a^	State	Geographical classification^b^	Living alone
1	62	Male	5 months	Subacute	QLD^c^	Metro	Yes
2	82	Female	3 years	Chronic	SA^d^	Regional	Yes
3	79	Female	8 months	Chronic	SA^d^	Metro	No
4	54	Male	4 months	Subacute	NSW^e^	Regional	Yes
5	57	Male	5 years	Chronic	VIC^f^	Metro	Yes
6	44	Male	3.5 months	Subacute	SA^d^	Regional	No
7	28	Female	6 months	Subacute	NSW^e^	Metro	No
8	47	Female	4 years	Chronic	QLD^c^	Regional	No

^a^ As defined by Bernhardt et al. [19] based on time since stroke.

^b^ Based on the Australian Statistical Geography Standard (ASGS)—Remoteness Area framework [20].

^c^ Queensland.

^d^ South Australia.

^e^ New South Wales.

^f^ Victoria.

The time since stroke ranged from 3.5 months to 5 years, with callers either in the chronic (*n* = 4) or subacute stage of recovery (*n* = 4). Callers were from four different states in Australia, with callers located in either a metro area (*n* = 4) or a regional area (*n* = 4) at the time of the call. None of the participants identified themselves as being Aboriginal or Torres Strait Islander (Table 1).

### 3.2 Thematic Analysis

**Figure 2:**
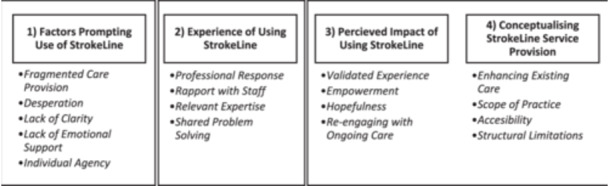
Themes and sub‐themes across the call process.

Four major themes were identified, including 17 sub‐themes. Key themes included (1) factors prompting use of StrokeLine, (2) experience of using StrokeLine, (3) perceived impact of using StrokeLine and (4) conceptualising StrokeLine service provision (Figure 2).

Theme 1 *Factors prompting use of StrokeLine*.

**[Sub‐theme: Fragmented care provision]** Participants discussed a lack of follow‐up from health professionals following discharge from hospital. Responses often pointed to very little direction given by healthcare professionals or other stroke services of what to do and where to go next. Participants also felt neglected by the healthcare system.

… I spent about an hour and a half with the specialist and that's it. That was the end of my care in relation to my stroke. So there was no follow‐up. They gave me a mobile phone number to call the stroke clinic and made it very clear that if I ever had any questions, any concerns, I was to call that number, and if I didn't get through, I was to leave a message and they would get back to me. I must have rung that number 10, 15 times and never had a response. No one's ever picked it up, and no one has ever returned the phone call.

(P6, male, subacute)

Participants, however, noted receiving an information pack when discharged from the hospital. Most called StrokeLine only after finding the number for the service in an information pack (such as MyStroke Journey) after some time had passed once they were home.

The only information they gave me at the hospital when I left was a leaflet.

(P6, male, subacute)

**[Sub‐theme: Desperation]** Participants often contacted StrokeLine as a ‘last resort’, only after exhausting all other known avenues of seeking solutions to their problems or after no longer knowing where to turn.

… and it was a call of last resort because I still can't work out who I'm meant to talk to. I have no mechanism of getting in contact with anyone that can provide me with advice at all.

(P6, male, subacute)

I think I rang on both occasions because I had exhausted my own mental and psychological resources—that's the point that I was at to make that phone call.

(P5, male, chronic)

**[Sub‐theme: Lack of clarity]** Participants contacted StrokeLine seeking clarity around their concerns.

Concerns themselves were often complex, compounded by conflicting information, advice and support needs. As such, there was never a singular reason for contacting the service.

I felt that I wasn't getting enough information from the medical profession that I felt made sense for me. And I'm one of these people, that I'm on a need to know basis. If I understand as best one can what's happening for you, then you can manage the situation. But if you don't know what's happening, you can't manage it because it's sort of a blind spot. And so to me it was logical because it was sort of to do with the stroke … that they were the logical people to perhaps give me some answers or at least point me in a direction that will be useful in terms of managing what was happening for me at the time.

(P1, female, chronic)

**[Sub‐theme: Seeking emotional support]** Along with seeking clarity and practical guidance, StrokeLine calls were often driven by participants' emotional states. Often, calls were prompted with no real direction and very limited expectations, as participants sought out the service to make sense of their changed circumstances, under heightened emotional distress.

I was quite keen to talk to someone who might have some level of understanding of what I've experienced and what is normal and what isn't normal.

(P6, male, subacute)

**[Sub‐theme: Individual agency]** All participants revealed individual agency in their active involvement in seeking control of their current situation, prompting them to call StrokeLine and initiate continuity of their own care.

It wasn't until it became quite evident that there was no support whatsoever in the process that I had experienced that I thought, well, I better be proactive here and I rang the StrokeLine.

(P6, male, subacute)

Theme 2 *Experience of using StrokeLine.*

**[Sub‐theme: Professional response]** Participants valued the way in which StrokeLine responded to their first contact. In particular, participants appreciated the timely and professional response of StrokeLine staff, even when their call was not answered on the first try.

So, I rang up and there was no one to answer my call and I left a message. And on both occasions the person got back really quickly. Like impressively quickly, as in when you leave a message you rarely get a call back in any … and on the first occasion—I got a call back from the lady, from one of them. And then another one of the ladies called back at the same time and I said, ‘Well, I think I'm already talking …’ The response was impressive and the response was impressive like twice over.

(P6, male, subacute)

**[Sub‐theme: Rapport with staff]** Participants experienced good rapport with StrokeLine staff during their contact. They felt comfortable sharing sensitive information about their experiences and perceived a sense of familiarity when interacting with StrokeLine staff. Most participants emphasised how they did not feel patronised when sharing their concerns and instead felt listened to.

I didn't feel like I was ringing up and there was a script that was being followed to provide me with guidance. I felt it was quite personal experience to what I experienced and it felt like … so the effectiveness of it, I think it was quite effective from that perspective.

(P6, male, subacute)

**[Sub‐theme: Relevant expertise]** Participants not only understood StrokeLine staff to be experienced enough to provide advice based on their professional qualifications and expertise but also appreciated that they had knowledge relevant to all aspects of their stroke care.

I think the balance that you've got with the people that you have on the stroke hotline for me was ideal. It wasn't someone who had had a stroke, but I felt that they had—well, maybe they have, I have no idea, I couldn't tell and they were able to provide me with caring advice.

(P6, male, subacute)

**[Sub‐theme: Shared problem‐solving]** Participants appreciated the way in which their concerns were addressed by StrokeLine staff, particularly in being included in the process and not simply being given the answers someone else thought they needed.

… she gave me direction and a sense of purpose. I think which was … there was another person saying, ‘I now have heard what you're experiencing and I think that what you're experiencing warrants you should go and see your GP and you should go and take this course of action’. And I think that was an important thing to gain from that conversation, which I haven't got anywhere else, not even from the GP when I talk to them.

(P6, male, subacute)

Theme 3 *Perceived impact of using StrokeLine.*

**[Sub‐theme: Validation of experience]** Participants felt that their experiences after having a stroke were validated and that there was value placed on the significance of their changed circumstances. In doing so, most participants felt that they could allow themselves to better accept what had happened.

She accepted what I said—she didn't sort of dismiss it as being trivial or not consequential. So for me, that was really good being confirmed in that way, that I had concerns that really needed to be looked at seriously, which was good.

(P1, female, chronic)

**[Sub‐theme: Empowerment]** Participants felt empowered after contacting StrokeLine and felt better able to take charge of their care. They perceived that their contact with StrokeLine equipped them with the skills that they needed to be able to do things themselves.

… and as a result … I listened to some podcasts, I downloaded books, and then I felt like I had more control about the experience that I was having.

(P6, male, subacute)

**[Sub‐theme: Hopefulness]** Participants felt hopeful after their contact with StrokeLine, particularly in feeling like they could now get through the difficulties that they experienced from stroke.

I'm trying to regain as much independence as I can, and people keep telling me how well I'm doing. And I think, you know, yes I have come a long way.

(P1, female, chronic)

So there was that little glimmer of hope that things might go a different way and I might be able to go back to getting the therapy that I felt I felt justified in asking for.

(P6, male, subacute)

**[Sub‐theme: Re‐engaging with ongoing care]** Participants felt motivated to take appropriate action in response to their needs, noting that their contact with StrokeLine prompted them to better engage with their post‐stroke recovery.

But they gave me the confidence to actually move on and say, okay, this needs other attention.

(P1, female, chronic)

I still felt overwhelmed about everything but I suppose there was that little bit of me that also was thinking that there is some light there and ‘I’ need to do something about it. So that was the thing. And I've learned that everybody has to fight for themselves. So nobody's going to knock on my door and say, ‘Hey, come and be part of the acquired brain injury unit.’ You've got to go out there and I suppose pipe yourself.

(P6, male, subacute)

Theme 4 *Conceptualising StrokeLine service provision.*

**[Sub‐theme: Enhancing existing care]** Participants noted that they did not know what to expect when first calling StrokeLine. After the call, however, participants understood the service to be valuable in enhancing their existing care and they would use it again.

As I said I've engaged with the process on two occasions and I guess they've sort of like been little stable points in quite a confusing, disjointed process where I guess I needed that contact at those times and have been aware since the first time that if I needed that I could reach out again and obviously get a great response. Not with an expectation that all of my answers would be there but there would be someone to listen to what I had experienced and give me some advice even if it's advice that I was aware of—just like having someone else tell you that this is something that you should do is quite powerful on occasions, you might know where the resources are, you might know what the prudent thing might be to do—like going visit your GP—but until someone articulates that and says it out loud, it's like maybe you don't get around to doing it.

(P6, male, subacute)

StrokeLine performed better than any other part of this system that I've stepped into.

(P6, male, subacute)

**[Sub‐theme: Scope of practice]** Participants understood that StrokeLine staff were unable to provide them with aspects of care that were (1) professionally outside the scope of practice of StrokeLine staff and (2) outside the scope of telehealth‐based service provision offered by StrokeLine.

The lass [female] I spoke to was very good at listening and asking me questions that I felt were really relevant. And although I didn't expect a diagnosis—obviously they're not in that sort of category. She did point me into a direction, like she did say to me, you probably need to make an appointment with a neurologist.

(P1, female, chronic)

I think I have a better appreciation of what can be offered having contacted them twice. I know even though I would love someone to be able to sort out the hassles I've got with the bureaucracy around the medical system, I know that they are not able to do that …

(P6, male, subacute)

**[Sub‐theme: Accessibility]** Participants appreciated the value of StrokeLine existing as a telehealth‐based service, particularly with regard to the immediate availability of support from the service and the ability to improve access to care for people living in areas with limited post‐stroke services.

You can just call the number and they're there.

(P8, female, subacute)

Well, I think accessibility and particularly, as I said previously, for regional people—and rural people would have the same issue. And I think it's a huge issue because I was talking to someone this morning and things are still [capital city]‐centric. Nothing happens beyond our [capital city]. And so I think being aware that there are people out there in remoter areas that really would find a service like this useful.

(P1, female, chronic)

**[Sub‐theme: Resource limitations]** All callers understood there to be potential resource limitations with the StrokeLine service, particularly around staffing, that led to delays in response time. Feedback around effectiveness of service provision consequently centred around the preference for calls to be answered on the first try, suggesting the need for improvement in this area.

I felt frustrated that I had to leave a voicemail at first. I understand though, that's because they can't answer everyone's calls and there's a lot of people that call. But I think the actual service is very good and effective … The one thing I found tricky was the leaving the voicemail and then also having to wait … sometimes you might call because you're feeling something right in that moment, and you might want support right then. And then they call back when they're free an hour later or whenever it is, and you might not want to talk about it anymore … the emotions are still there, you might just not want to speak about it then.

(P8, female, subacute)

They need to revamp their answering machine because it leaves you with the feeling there's no one there. It's the way it's expressed. It needs to say something like ‘all our professional workers are busy at the moment’ rather than saying ‘there's no one here’ or sort of thing and you're left with the feeling of ‘should I ring back or not?’

(P1, female, chronic)

## 4 Discussion

Stroke survivors were prompted to use the StrokeLine service after experiencing fragmented care provision in their post‐stroke recovery and feeling abandoned by the healthcare system. Contact with StrokeLine was often initiated out of desperation and perceived as a last resort when other avenues of seeking advice and emotional support were exhausted. In contacting StrokeLine, all participants revealed individual agency in their active involvement in seeking continuity of care. Participants reported an overall positive experience while using StrokeLine and appreciated the professional response and expertise of StrokeLine staff during the call. Stroke survivors felt comfortable sharing their concerns with staff and valued being involved in the process of helping them find practical solutions.

After using StrokeLine, participants perceived a positive impact on their stroke journey. They felt that their experiences were validated, motivating and empowering them to take charge of their recovery. Participants felt a renewed sense of hopefulness and noted that they would use StrokeLine again if needed, as they now knew what they could expect from the service. Stroke survivors were also able to form a better conceptualisation of StrokeLine service provision after the call. They understood the value of the service to enhance existing care. Further, they understood that the service was limited by both the scope of practice of StrokeLine staff and the telehealth‐based model of care under which the service functions. Participants, however, also understood the potential strength of the service in providing timely access to support in geographical areas with limited post‐stroke services. Finally, when suggesting recommendations for improving the StrokeLine service, all participants perceived structural limitations around resource allocation and staffing. Consequently, most recommendations for improvement were believed unlikely to be actioned if these perceived limitations to resource allocation were not first resolved. Recommendations for service improvement included (i) increasing the awareness and promotion of the service, (ii) increasing the likelihood of answering calls in the first instance and (iii) providing a more personable voicemail message if this could not be achieved. Furthermore, another recommendation is increasing awareness of StrokeLine for stroke survivors.

It is important to note that no participants in this study called StrokeLine during the acute stage of their recovery. This may be reflective of findings from a study reporting that stroke survivors often do not know what to ask during the early stages of recovery and rely on health professionals to provide them with appropriately selected information [25]. Participants in this study, however, noted receiving very limited care provision after hospital discharge and even later in their stroke recovery. Most participants received an information pack when discharged from hospital in which they later found the StrokeLine number. Numerous studies have highlighted the importance of providing information ‘just in time’ to facilitate the self‐management of care in stroke [5, 26]. If information is provided at a time when a person is not ready to take ownership of their condition, most often, people are left feeling overwhelmed and abandoned by their healthcare providers [27]. As such, self‐management of care is contingent on people revealing individual agency and seeking to take active control in the continuity of their care [28]. Helpline interactions, therefore, are able to reinforce self‐management by providing timely support, relevant information and empowering callers when the caller chooses to engage [29].

In the current study, concerns discussed during the call were often multilayered and there was never a singular, or definitive, reason for calling StrokeLine. Information, advice and support were often all sought during the same call and callers were unable to distinctly differentiate between what was offered to them. Interactions of a similar nature in helpline‐based care provision are discussed extensively in the research. Arvidsson et al. identified that understanding the precise description of a caller's problem when they called a rheumatology telephone helpline was difficult [13]. Initially characterising the caller's experience by building a feeling of mutual trust and solidarity, however, later aided in clarifying the explicit reason for the call [13]. As such, the strength of telephone‐based service provision lies in the call‐taker's ability to combine emotional support with practical information [30].

Findings from a systematic review suggest that interview studies do not necessarily require large sample sizes to reach saturation. In particular, a homogeneous study cohort may require a smaller sample size to reach saturation. This may be achieved through nine to 17 interviews. This is more likely to occur within an objectively defined scope of exploration where the data are collected rigorously [31]. As such, the small sample size reported in this study may be adequate to provide a rich account of people's experiences.

### 4.1 Strengths and Limitations

The interview process allowed participants to express their stroke experiences in a continuum before, during and after their contact with StrokeLine. Understanding the wider context of factors prompting stroke survivors to call StrokeLine highlights the gaps in current provision of care in stroke and could provide insight into understanding the longer term needs of stroke survivors. This study was also guided by partnership with the Stroke Foundation in Australia, allowing findings from the study to support timely feedback for service improvement and relevant reporting to policymakers.

There were limitations to study recruitment that may have affected the sample size reported in this study. The majority of recruitment occurred during the COVID‐19 pandemic and relied heavily on StrokeLine staff who were located in Melbourne, Australia—a city that experienced the world's longest lockdown period. During this time, it is likely that StrokeLine staff had competing priorities that led to a long period of recruitment for this study. In turn, limited data were available on the use of StrokeLine during the recruitment period. Between November 2019 and November 2020, however, StrokeLine received 429 calls from stroke survivors [32]. Stroke survivors with a more positive experience of StrokeLine service provision are more likely to have expressed an interest in being interviewed and as such, experiences that did not meet the caller's expectations may not have been captured in this study.

The level of post‐stroke impairment of each participant was also not explicitly captured during the interview process and the experiences and perceived impact of using StrokeLine in severe stroke versus mild stroke may differ. Users of the StrokeLine service who self‐reported as having had a stroke were included in this study; future studies could include participants who have clinician‐ or medical record–confirmed stroke to further validate findings. Although not an exclusion criterion, no stroke survivors with communication difficulties expressed an interest in participating in the study. All participants were also from European backgrounds and findings may not be inclusive of the views of people from other cultural backgrounds. Future studies could incorporate StrokeLine service users from more diverse backgrounds to identify if changes could be made to improve service delivery to broader populations.

### 4.2 Recommendations

Future research should seek to explore the definition of information, support and advice in stroke care to understand how and when to best provide each across the continuum of stroke recovery and to better match the expectations of stroke survivors. Further research should also seek to understand how best to integrate services (such as StrokeLine) within existing health systems at an appropriate time within the stroke illness trajectory. Better understanding factors prompting stroke survivors to reveal individual agency to take control of their care continuity may provide insight into when to best have services available to support their self‐management. There is also a need to better understand factors of nonparticipation and low engagement with StrokeLine. In a previous study, almost 40% of StrokeLine users were reported as carers and future research should also explore their experiences of using the service [32].

In response to the COVID‐19 pandemic, the relevance of telehealth‐based care provision and a focus on value‐centred healthcare redesign warrants an economic evaluation of StrokeLine service provision. As such, this study highlights implications for policymakers in supporting the future sustainability of services such as StrokeLine.

## 5 Conclusion

Stroke survivors used a stroke helpline in response to fragmented care provision in other settings and after using the service, most perceived a positive impact on their stroke recovery. Participants felt empowered and motivated to re‐engage with their ongoing care. Stroke survivors also understood StrokeLine to be able to enhance their existing care. Stroke survivor awareness of the StrokeLine service could be improved. Structural limitations were perceived as the main driver for the delayed response time in answering calls in the first instance.

## Author Contributions

**Muneeba T. Chaudhry:** conceptualisation, methodology, data curation, writing–original draft, writing–review and editing, investigation. **Alana B. McCambridge:** conceptualisation, methodology, data curation, investigation, writing–original draft, writing–review and editing, supervision, formal analysis. **Esminio I.I. Rivera:** formal analysis, writing–review and editing. **Scott William:** writing–review and editing, project administration. **Peter Stubbs:** writing–review and editing, supervision. **Arianne Verhagen:** conceptualisation, methodology, supervision, investigation, formal analysis, writing–original draft, writing–review and editing, resources. **Caleb Ferguson:** conceptualisation, methodology, supervision, data curation, formal analysis, investigation, writing–original draft, writing–review and editing, resources, project administration.

## Conflicts of Interest

C.F. was a member of the Stroke Foundation (Australia) Research Advisory Committee (2016‐2022).

## Data Availability

Due to the sensitive nature of the data collected for this study, requests to access the data set from qualified researchers trained in human subject confidentiality protocols may be sent to the corresponding author.
